# Supplementary material for: Estimating the potential impact of global research on neglected tropical diseases on population-level indicators of health access, sanitation, and research capacity
Source: PLoS Negl Trop Dis. 2026 May 21;20(5):e0014343. doi: 10.1371/journal.pntd.0014343 (PMC13218535; doi:10.1371/journal.pntd.0014343)
Supplement: S1 File — Search strategy in databases. (DOCX) [file pntd.0014343.s001.docx]

**Supplementary Material #1**

Search strategies used according to databases:

***Scopus database***

TITLE("Neglected Diseases") OR TITLE("Neglected Disease") OR TITLE("Neglected Tropical Diseases") OR TITLE("Neglected Tropical Disease") OR TITLE("Lymphatic Filariasis") OR TITLE("Filarial Elephantiases") OR TITLE("Filarial Elephantiasis") OR TITLE("Lymphatic Filariases") OR TITLE("Lymphatic Filariasis") OR TITLE("Bancroftian Elephantiases") OR TITLE("Wuchereria bancrofti Infection") OR TITLE("Bancroftian Filariasis") OR TITLE("Bancroftian Filariases") OR TITLE("Bancroftian Elephantiasis") OR TITLE("Malayi Filariasis") OR TITLE("Malayi Filariases") OR TITLE("Brugian Filariasis") OR TITLE("Brugian Filariases") OR TITLE("Malayi Elephantiases") OR TITLE("Malayi Elephantiasis") OR TITLE(Onchocerciasis) OR TITLE(Schistosomiasis) OR TITLE(Schistosomiases) OR TITLE("Schistoma Infection") OR TITLE("Schistoma Infections") OR TITLE(Bilharziasis) OR TITLE(Bilharziases) OR TITLE("Katayama Fever") OR TITLE(Helminthiasis) OR TITLE(Helminthiases) OR TITLE("Nematomorpha Infection") OR TITLE("Nematomorpha Infections") OR TITLE(Trachoma) OR TITLE("Egyptian Ophthalmia") OR TITLE(Leprosy) OR TITLE("Hansen Disease") OR TITLE("Hansen's Disease") OR TITLE(Dracunculiasis) OR TITLE(Dracunculosis) OR TITLE(Dracunculoses) OR TITLE("Guinea Worm Disease") OR TITLE("Guinea Worm Diseases") OR TITLE("Guinea Worm Infection") OR TITLE(Yaws) OR TITLE("Frambesia Tropica") OR TITLE("Frambesia Tropicas") OR TITLE(Frambesia) OR TITLE(Frambesias) OR TITLE("Human African Trypanosomiasis") OR TITLE("Chagas Disease") OR TITLE("Trypanosoma cruzi Infection") OR TITLE("Trypanosoma cruzi Infections") OR TITLE("Chagas' Disease") OR TITLE("South American Trypanosomiasis") OR TITLE("American Trypanosomiasis") OR TITLE("Visceral Leishmaniasis") OR TITLE("Kala-Azar") OR TITLE("Kala Azar") OR TITLE("Black Fever") OR TITLE(Rabies) OR TITLE("Encephalitic Rabies") OR TITLE("Soil-Transmitted Helminthiases") OR TITLE("Buruli Ulcer") OR TITLE("Mycobacterium ulcerans Infections") OR TITLE("Mycobacterium ulcerans Infection") OR TITLE(Dengue) OR TITLE("Break-Bone Fever") OR TITLE("Break Bone Fever") OR TITLE("Breakbone Fever") OR TITLE(Chikungunya) OR TITLE("Chikungunya Fever") OR TITLE("Chikungunya Virus Infection") OR TITLE("Chikungunya Virus Infections") OR TITLE(Echinococcosis) OR TITLE("Echinococcus Infection") OR TITLE("Echinococcus Infections") OR TITLE("Echinococcus granulosus Infection") OR TITLE("Echinococcus granulosus Infections") OR TITLE("Foodborne Trematodiases") OR TITLE("Cutaneous Leishmaniasis") OR TITLE("Cutaneous Leishmaniases") OR TITLE("American Leishmaniasis") OR TITLE("Oriental Sore") OR TITLE(Mycetoma) OR TITLE(Maduromycosis) OR TITLE(Eumycetoma) OR TITLE(Actinomycetoma) OR TITLE(Chromoblastomycosis) OR TITLE(Chromomycosis) OR TITLE(Chromomycoses) OR TITLE(Scabies) OR TITLE("Sarcoptic Mange") OR TITLE("Snakebite Envenoming") OR TITLE("Snake Bite") OR TITLE(Snakebites) OR TITLE(Snakebite) OR TITLE("Snake Envenomings") OR TITLE("Snakebite Envenomation") OR TITLE("Snakebite Envenomations") OR TITLE("Snake Envenomations") OR TITLE(Taeniasis) OR TITLE(Taeniases) OR TITLE("Taenia Infection") OR TITLE("Taenia Infections") OR TITLE("Taenia glomeratus Infection") OR TITLE("Taenia solium Infection") OR TITLE("Taenia solium Infections") OR TITLE("Taenia serialis Infections") OR TITLE("Taenia multiceps Infection") OR TITLE("Taenia brauni Infection") OR TITLE(Cysticercosis) OR TITLE(Cysticercoses) OR TITLE(Coenurosis) OR TITLE(Coenuroses) OR TITLE("Coenuri Infection") OR TITLE("Coenuri Infections") OR TITLE("Coenurus Infection") OR TITLE("Coenurus Infections") OR TITLE("Coenurus cerebralis Infection") OR TITLE("Coenurus cerebralis Infections") OR TITLE("Cysticercus cellulosae Infection") OR TITLE("Cysticercus cellulosae Infections") OR TITLE("Taenia solium Cysticercosis") OR TITLE("Taenia solium Cysticercoses") = 143,475 documents found.

***PubMed database***

(((((((((((((((((((((((((((((((((((((((((((((((((((((((((((((((((((((((((((((((((((((((((((((((((((((((((((((((((((((((((((("Neglected Diseases"[Title]) OR ("Neglected Disease"[Title])) OR ("Neglected Tropical Diseases"[Title])) OR ("Neglected Tropical Disease"[Title])) OR ("Lymphatic Filariasis"[Title])) OR ("Filarial Elephantiases"[Title])) OR ("Filarial Elephantiasis"[Title])) OR ("Lymphatic Filariases"[Title])) OR ("Lymphatic Filariasis"[Title])) OR ("Bancroftian Elephantiases"[Title])) OR ("Wuchereria bancrofti Infection"[Title])) OR ("Bancroftian Filariasis"[Title])) OR ("Bancroftian Filariases"[Title])) OR ("Bancroftian Elephantiasis"[Title])) OR ("Malayi Filariasis"[Title])) OR ("Malayi Filariases"[Title])) OR ("Brugian Filariasis"[Title])) OR ("Brugian Filariases"[Title])) OR ("Malayi Elephantiases"[Title])) OR ("Malayi Elephantiasis"[Title])) OR ("Onchocerciasis"[Title])) OR ("Schistosomiasis"[Title])) OR ("Schistosomiases"[Title])) OR ("Schistoma Infection"[Title])) OR ("Schistoma Infections"[Title])) OR ("Bilharziasis"[Title])) OR ("Bilharziases"[Title])) OR ("Katayama Fever"[Title])) OR ("Helminthiasis"[Title])) OR ("Helminthiases"[Title])) OR ("Nematomorpha Infection"[Title])) OR ("Nematomorpha Infections"[Title])) OR ("Trachoma"[Title])) OR ("Egyptian Ophthalmia"[Title])) OR ("Leprosy"[Title])) OR ("Hansen Disease"[Title])) OR ("Hansen's Disease"[Title])) OR ("Dracunculiasis"[Title])) OR ("Dracunculosis"[Title])) OR ("Dracunculoses"[Title])) OR ("Guinea Worm Disease"[Title])) OR ("Guinea Worm Diseases"[Title])) OR ("Guinea Worm Infection"[Title])) OR ("Yaws"[Title])) OR ("Frambesia Tropica"[Title])) OR ("Frambesia Tropicas"[Title])) OR ("Frambesia"[Title])) OR ("Frambesias"[Title])) OR ("Human African Trypanosomiasis"[Title])) OR ("Chagas Disease"[Title])) OR ("Trypanosoma cruzi Infection"[Title])) OR ("Trypanosoma cruzi Infections"[Title])) OR ("Chagas' Disease"[Title])) OR ("South American Trypanosomiasis"[Title])) OR ("American Trypanosomiasis"[Title])) OR ("Visceral Leishmaniasis"[Title])) OR ("Kala-Azar"[Title])) OR ("Kala Azar"[Title])) OR ("Black Fever"[Title])) OR ("Rabies"[Title])) OR ("Encephalitic Rabies"[Title])) OR ("Soil-Transmitted Helminthiases"[Title])) OR ("Buruli Ulcer"[Title])) OR ("Mycobacterium ulcerans Infections"[Title])) OR ("Mycobacterium ulcerans Infection"[Title])) OR ("Dengue"[Title])) OR ("Break-Bone Fever"[Title])) OR ("Break Bone Fever"[Title])) OR ("Breakbone Fever"[Title])) OR ("Chikungunya"[Title])) OR ("Chikungunya Fever"[Title])) OR ("Chikungunya Virus Infection"[Title])) OR ("Chikungunya Virus Infections"[Title])) OR ("Echinococcosis"[Title])) OR ("Echinococcus Infection"[Title])) OR ("Echinococcus Infections"[Title])) OR ("Echinococcus granulosus Infection"[Title])) OR ("Echinococcus granulosus Infections"[Title])) OR ("Foodborne Trematodiases"[Title])) OR ("Cutaneous Leishmaniasis"[Title])) OR ("Cutaneous Leishmaniases"[Title])) OR ("American Leishmaniasis"[Title])) OR ("Oriental Sore"[Title])) OR ("Mycetoma"[Title])) OR ("Maduromycosis"[Title])) OR ("Eumycetoma"[Title])) OR ("Actinomycetoma"[Title])) OR ("Chromoblastomycosis"[Title])) OR ("Chromomycosis"[Title])) OR ("Chromomycoses"[Title])) OR ("Scabies"[Title])) OR ("Sarcoptic Mange"[Title])) OR ("Snakebite Envenoming"[Title])) OR ("Snake Bite"[Title])) OR ("Snakebites"[Title])) OR ("Snakebite"[Title])) OR ("Snake Envenomings"[Title])) OR ("Snakebite Envenomation"[Title])) OR ("Snakebite Envenomations"[Title])) OR ("Snake Envenomations"[Title])) OR ("Taeniasis"[Title])) OR ("Taeniases"[Title])) OR ("Taenia Infection"[Title])) OR ("Taenia Infections"[Title])) OR ("Taenia glomeratus Infection"[Title])) OR ("Taenia solium Infection"[Title])) OR ("Taenia solium Infections"[Title])) OR ("Taenia serialis Infections"[Title])) OR ("Taenia multiceps Infection"[Title])) OR ("Taenia brauni Infection"[Title])) OR ("Cysticercosis"[Title])) OR ("Cysticercoses"[Title])) OR ("Coenurosis"[Title])) OR ("Coenuroses"[Title])) OR ("Coenuri Infection"[Title])) OR ("Coenuri Infections"[Title])) OR ("Coenurus Infection"[Title])) OR ("Coenurus Infections"[Title])) OR ("Coenurus cerebralis Infection"[Title])) OR ("Coenurus cerebralis Infections"[Title])) OR ("Cysticercus cellulosae Infection"[Title])) OR ("Cysticercus cellulosae Infections"[Title])) OR ("Taenia solium Cysticercosis"[Title])) OR ("Taenia solium Cysticercoses"[Title])) = 121,748 documents found.

***WoS database***

(TI="Neglected Diseases" OR TI="Neglected Disease" OR TI="Neglected Tropical Diseases" OR TI="Neglected Tropical Disease" OR TI="Lymphatic Filariasis" OR TI="Filarial Elephantiases" OR TI="Filarial Elephantiasis" OR TI="Lymphatic Filariases" OR TI="Lymphatic Filariasis" OR TI="Bancroftian Elephantiases" OR TI="Wuchereria bancrofti Infection" OR TI="Bancroftian Filariasis" OR TI="Bancroftian Filariases" OR TI="Bancroftian Elephantiasis" OR TI="Malayi Filariasis" OR TI="Malayi Filariases" OR TI="Brugian Filariasis" OR TI="Brugian Filariases" OR TI="Malayi Elephantiases" OR TI="Malayi Elephantiasis" OR TI=Onchocerciasis OR TI=Schistosomiasis OR TI=Schistosomiases OR TI="Schistoma Infection" OR TI="Schistoma Infections" OR TI=Bilharziasis OR TI=Bilharziases OR TI="Katayama Fever" OR TI=Helminthiasis OR TI=Helminthiases OR TI="Nematomorpha Infection" OR TI="Nematomorpha Infections" OR TI=Trachoma OR TI="Egyptian Ophthalmia" OR TI=Leprosy OR TI="Hansen Disease" OR TI="Hansen's Disease" OR TI=Dracunculiasis OR TI=Dracunculosis OR TI=Dracunculoses OR TI="Guinea Worm Disease" OR TI="Guinea Worm Diseases" OR TI="Guinea Worm Infection" OR TI=Yaws OR TI="Frambesia Tropica" OR TI="Frambesia Tropicas" OR TI=Frambesia OR TI=Frambesias OR TI="Human African Trypanosomiasis" OR TI="Chagas Disease" OR TI="Trypanosoma cruzi Infection" OR TI="Trypanosoma cruzi Infections" OR TI="Chagas' Disease" OR TI="South American Trypanosomiasis" OR TI="American Trypanosomiasis" OR TI="Visceral Leishmaniasis" OR TI="Kala-Azar" OR TI="Kala Azar" OR TI="Black Fever" OR TI=Rabies OR TI="Encephalitic Rabies" OR TI="Soil-Transmitted Helminthiases" OR TI="Buruli Ulcer" OR TI="Mycobacterium ulcerans Infections" OR TI="Mycobacterium ulcerans Infection" OR TI=Dengue OR TI="Break-Bone Fever" OR TI="Break Bone Fever" OR TI="Breakbone Fever" OR TI=Chikungunya OR TI="Chikungunya Fever" OR TI="Chikungunya Virus Infection" OR TI="Chikungunya Virus Infections" OR TI=Echinococcosis OR TI="Echinococcus Infection" OR TI="Echinococcus Infections" OR TI="Echinococcus granulosus Infection" OR TI="Echinococcus granulosus Infections" OR TI="Foodborne Trematodiases" OR TI="Cutaneous Leishmaniasis" OR TI="Cutaneous Leishmaniases" OR TI="American Leishmaniasis" OR TI="Oriental Sore" OR TI=Mycetoma OR TI=Maduromycosis OR TI=Eumycetoma OR TI=Actinomycetoma OR TI=Chromoblastomycosis OR TI=Chromomycosis OR TI=Chromomycoses OR TI=Scabies OR TI="Sarcoptic Mange" OR TI="Snakebite Envenoming" OR TI="Snake Bite" OR TI=Snakebites OR TI=Snakebite OR TI="Snake Envenomings" OR TI="Snakebite Envenomation" OR TI="Snakebite Envenomations" OR TI="Snake Envenomations" OR TI=Taeniasis OR TI=Taeniases OR TI="Taenia Infection" OR TI="Taenia Infections" OR TI="Taenia glomeratus Infection" OR TI="Taenia solium Infection" OR TI="Taenia solium Infections" OR TI="Taenia serialis Infections" OR TI="Taenia multiceps Infection" OR TI="Taenia brauni Infection" OR TI=Cysticercosis OR TI=Cysticercoses OR TI=Coenurosis OR TI=Coenuroses OR TI="Coenuri Infection" OR TI="Coenuri Infections" OR TI="Coenurus Infection" OR TI="Coenurus Infections" OR TI="Coenurus cerebralis Infection" OR TI="Coenurus cerebralis Infections" OR TI="Cysticercus cellulosae Infection" OR TI="Cysticercus cellulosae Infections" OR TI="Taenia solium Cysticercosis" OR TI="Taenia solium Cysticercoses") = 48,538 documents found.
