# Supplementary material for: Estimating the potential impact of global research on neglected tropical diseases on population-level indicators of health access, sanitation, and research capacity
Source: PLoS Negl Trop Dis. 2026 May 21;20(5):e0014343. doi: 10.1371/journal.pntd.0014343 (PMC13218535; doi:10.1371/journal.pntd.0014343)
Supplement: Attachment — Submitted filename: Manuscript ID PNTD-D-25-01315 - Answer to reviewers.docx [file pntd.0014343.s008.docx]

**Answer to reviewers**

**Manuscript ID PNTD-D-25-01315**

**Measuring the Potential Impact of Global Research on Neglected Tropical Diseases on Population-Level Indicators of Health Access, Sanitation, and Research Capacity**

*We are grateful with the Editor and Reviewers for the expert reading of the manuscript. We have found the comments appropriate and helpful. They have permitted us to improve the manuscript. Appropriate changes were made and highlighted in the revised manuscript according to suggestions. Following are the responses to the comments.*

**Editor**

The reviews for this manuscript are generally positive and highlight the impressive scope and need for this study. Reviewers have brought up a few issues to be addressed prior to publication. We hope that you will consider revising the manuscript following reviewers’ comments.

***r/ We sincerely thank the Editor for the positive assessment and the opportunity to improve our work. We have carefully addressed all the reviewers' comments in this revised manuscript***

***Methods***

**Reviewer #3**

**I suggest that hypotheses and expectations are more clearly described in the introduction. For example what do you expect for each income group and for each indicator? At this time, it feels like an overwhelming set of indicators being used with no clear justification as to why each specific one needs to be included.**

*r/ We thank the reviewer for this suggestion. We have revised the end of the Introduction to explicitly state our expectations and the hypothesis we aimed to test.*

**There’s a large difference between the output from a narrative vs systematic review. I’m not convinced you should have them put in the same category. I suggest you also explicitly describe what was done to harmonize variables because the harmonization processes may not be agreeable to all readers.**

*r/ We thank the reviewer for this valid point. We agree that systematic and narrative reviews differ significantly in scope and evidentiary value. However, the core analysis of this manuscript evaluates the association between the aggregate volume of scientific production and population indicators, rather than isolating the effects of specific article types.*

*Since the distinction between review types does not affect our regression models (which utilize total publication counts), we have removed the section detailing this specific grouping to avoid confusion. Furthermore, we have streamlined the Methods section by removing descriptions of variables not used in the final regression analysis (such as h-index normalization and citation processing). The revised methodology now focuses exclusively on the data processes relevant to the study's main aim, making the section clearer and easier to follow.*

**It will be helpful to have a table that clearly labels exclusion and inclusion criteria. Alignment with these criteria keeps coming up in sections of the paper and it’s helpful if the reader has a place to go where all of these are clearly listed.**

*r/ We thank the reviewer for this suggestion. The manuscript currently includes a flowchart detailing the document selection process, from the initial search results to the final articles included after screening, along with the specific reasons for exclusion (Figure 1). Additionally, the full search strategy and criteria can be consulted in Supplementary Material 1.*

**More justification is needed as to why some variables were categorized as response vs predictor vs moderator. Perhaps a flow chart or some method because right now it seems very subjective.**

*r/ We thank the reviewer for this comment. It is true that this categorization involves a degree of subjectivity. There is often no definitive reason to prefer a variable as independent over dependent, or vice versa, since both directions estimate different aspects of the same interaction. For example, studying the impact of research on a country's economy is just as relevant as studying the impact of the economy on the research it produces. In this context, we opted to assign roles based on theoretical coherence and interpretability, aligning with the specific relationships we aimed to explore in the manuscript. This selection criteria is now explicitly stated in the methodology.*

**Focusing on just physicians and nurses is ignoring the non-physicians (environmental health and veterinary medicine) who also conduct research in these types of fields so I think the picture may be incomplete here. At least mention this as a limitation in the discussion.**

*r/ We thank the reviewer for this insightful comment. We agree and have added this as a specific limitation in the Limitations section.*

**Can you elaborate on the definition of retrospective longitudinal analysis? I would categorize this study as a systematic literature review with a meta-analysis.**

*r/ We thank the reviewer for this opportunity to clarify our methodological approach. While we understand the inclination to categorize this as a systematic review and meta-analysis due to the use of literature databases and meta-analytical statistical tools, we respectfully maintain that 'Retrospective Longitudinal Analysis' is the most accurate definition for this study design.*

*Given that a systematic review typically aims to synthesize the findings or truth claims of individual studies to answer a clinical or theoretical question (e.g., 'Does Drug A work?'). In contrast, our study does not extract or synthesize the results of the 107,251 included papers. Instead, we treat the volume of scientific production itself as an independent or dependent variable and track its statistical association with real-world population indicators over time. Hence, this study is not an inductive summary of existing knowledge (a review). Rather, it is a deductive test of the falsifiable hypothesis that scientific effort drives societal improvement. We are observing the behavior of the scientific system over time, treating publications as objective data points in a longitudinal time series, rather than reviewing their content.*

*Therefore, while we utilize meta-analysis as a statistical technique to pool regression coefficients across income groups, the study design is fundamentally a longitudinal observation of the scientific ecosystem, not a review of the literature's conclusions. We believe maintaining this distinction is crucial for the reader to understand that we are measuring the impact of research activity, not summarizing what that research found.*

**Provide the dates of database search explicitly. So studies of any type whether empirical or theoretical or editorial were included? I realize you mention something along these lines later but more clarification is needed on the nature of studies pursued (what study designs did you consider).**

*r/ We thank the reviewer for highlighting this important point. The specific dates of the database search have now been explicitly added to the Methods section. Additionally, we have included the breakdown of articles by study design in the new Supplementary Material 2.*

**In the R code, there is annotation about low to high heterogeneity, which will be useful to have in the main text to understand your definitions.**

*r/ We thank the reviewer for this suggestion. We have now added these definitions to the Methods section of the manuscript.*

**Results**

**Reviewer #3**

**The results generally match the methods. I appreciate the supplemental files where I was able to see all the results.**

*r/* *We thank the reviewer for this positive assessment. We are glad that the supplementary files provided the necessary transparency and detail to support the results.*

**Please provide a summary of studies found - what you included and excluded and why and years/continents represented. It would be good to include countries where most pubs came from and the most common NTDs researched.**

*r/ We thank the reviewer for this suggestion. The flowchart in Figure 1 details the exclusion criteria and selection process. Additionally, we have created a new Supplementary Material 2, which presents the total number of articles categorized by country and document type.*

**Please also give a summary as to whether assumptions of using regression have been met.**

*r/ We thank the reviewer for this comment. We assessed the model fit and validity for all regressions using standard metrics (including R^2^, adjusted R^2^, and standard errors). The complete characteristics and full results for every linear model are provided in Supplementary Material 3.*

**Table 1: Please define how variable type was assigned. I suggest the table moves to supplement and include in a new Table 1 only the indicators that were used as non-moderators.**

*r/ We thank the reviewer for this suggestion. There is no definitive reason to prefer a variable as independent over dependent, or vice versa, since both directions estimate different aspects of the same interaction. In this context, we opted to assign roles based on theoretical coherence and interpretability, aligning with the specific relationships we aimed to explore in the manuscript. This selection criteria is now explicitly stated in the methodology. Additionally, we have moved the full table to the Supplementary Material, since the moderators that achieved significant results are already included in the subsequent tables.*

**Table 2: Why not p-values next to Q column here?**

*r/ We thank the reviewer for this suggestion. To maintain the table's readability, we have indicated the statistical significance of the Q statistic using asterisks directly in the column. Regarding the pooled coefficients, significance is established when the 95% confidence interval excludes zero. The exact p-values for all statistics are available in Supplementary Material 4.*

**Figure 1. Can you briefly state why were reports not retrieved? Just to ensure this didn’t add too much bias in the results. I suggest this figure moves to supplement. If it stays, it should go under results (not in methods).**

*r/ It was clarified that we were referring to results for which the full text could not be accessed.*

**Figure 2: Suggest switching the right column headings to vertical headings over each set of indicators so you can expand the heat map portion. Perhaps think about suitable ways of making some of those long indicators into shorter ones because it’s hard to read the heat map otherwise. Generally speaking being able to look at point estimates for each income group across each indicator would provide more information and align with the text better. Not sure why there's a white space for LIC R&D funding flows.**

*r/ We thank the reviewer for these observations. The right column with the indicator categories is now vertical. However, we consider it appropriate to maintain the full names of the indicators to avoid potential confusion. Additionally, we believe the current heatmap visualization is appropriate, given that its main purpose is to compare effects within income groups for each indicator, rather than to plot the raw coefficient numbers; these values are reported in the Results section and can also be consulted in Supplementary Material 3. Lastly, as noted in the figure legend, blank cells indicate insufficient data for analysis.*

**Can you provide a bar chart of number of pubs per year or something along those lines to give the reader insight as to the temporal trend?**

*r/ We thank the reviewer for this suggestion. While a bar chart would illustrate the temporal growth of the field, the primary objective of this study is to determine the statistical association between research output and population indicators, rather than to characterize the bibliometric trends themselves. Therefore, to maintain the focus on the regression and meta-analysis results, we have decided not to include this figure. However, the total number of publications stratified by income group is available in Supplementary Material 2.*

**It would also be useful if you had a choropleth global map showing where pubs are coming from.**

*r/ We thank the reviewer for this suggestion. While we agree that a choropleth map provides a valuable visual summary, the primary scope of this study is to evaluate the longitudinal association between research and population indicators, rather than to provide a descriptive bibliometric profile. To maintain the focus on the regression and meta-analysis results, we have opted not to include the map. However, the complete data on publication counts by country is available in Supplementary Material 2 for readers interested in the geographical distribution.*

**Conclusions**

**Reviewer #1**

**in the Discussion section: Expand the discussion on practical and policy implications, specifying how the findings can inform funding agencies, WHO initiatives, or national NTD programs.**

*r/ We thank the reviewer for this crucial suggestion. We have expanded the Discussion to include a specific reflection on policy implications for funding agencies and global health initiatives. This addition utilizes the concept of 'development coherence' (proposed by the authors in this work) to suggest that funders must shift their focus from 'capacity building' (increasing volume) to 'coherence building' (aligning research with local absorptive capacity).*

**Clarify that negative or non-significant results (e.g., absence of association with DALYs) do not indicate “no impact,” but rather a lack of statistical traceability. This distinction will help avoid possible misinterpretations.**

*r/ We thank the reviewer for this important point. We clarify that DALYs were not used in this specific analysis, as they belong to the 'Disease Burden and Infectious Diseases' domain, which is addressed in a separate manuscript. Consequently, we have removed the mention of DALYs from the Abstract, where it had been included in error.*

**Consider adding a final subsection on “policy translation”, including actionable recommendations such as strengthening local research capacity, promoting South–South collaborations, and encouraging implementation studies.**

*r/ We thank the reviewer for this helpful suggestion. We have added a final subsection titled 'Policy translation' to the Discussion. In this new section, we frame the actionable recommendations, specifically prioritizing implementation studies, strengthening local research capacity, and promoting South–South collaborations, within the concept of 'development coherence', a framework proposed by the authors in this manuscript to explain the structural requirements for research impact.*

**in the Limitation section:**

**Add a note about potential publication bias, since the most visible studies tend to come from high-income countries.**

*r/ We thank the reviewer for this important observation. We have explicitly added a note on publication bias to the Limitations section, acknowledging that reliance on major indexed databases tends to favor the visibility of research from high-income countries.*

**Specify that the use of annual, country-level averages implies a lack of temporal and spatial granularity, which could dilute local or short-term effects.**

*r/ We thank the reviewer for this observation. We respectfully submit that this level of aggregation is intrinsic to our study design, which specifically aims to identify macro-geographical patterns of 'scientific coherence' rather than to evaluate local, short-term intervention effects. By grouping data by World Bank income levels, our objective was to minimize noise from country-specific fluctuations and outliers, thereby allowing us to detect robust, structural associations between research ecosystems and development indicators that persist across economic strata. Consequently, we have maintained the current text, as this approach is central to the study's aim of assessing global systemic trends.*

**Suggest the future inclusion of policy output indicators (e.g., national health legislation or domestic funding for NTD research) to provide a more comprehensive view of research impact.**

*r/ We thank the reviewer for this insightful suggestion. We agree that policy output indicators are crucial for a holistic view of impact. We have added a statement to the Conclusions emphasizing that our methodological framework and open-access code are designed to be adaptable, specifically encouraging future studies to apply this method to policy domains such as national legislation and domestic funding.*

**Reviewer #3**

**I would suggest removing p and beta values from the discussion. You may want to use language that’s less statistical here. I think the most compelling results are WASH-related and should come up sooner.**

*r/ We thank the reviewer for this suggestion. Given the considerable volume of results, we considered it appropriate to mention the p-values and coefficients in the text to facilitate reading; this ensures the reader is not obliged to look back to verify the effect magnitude. Additionally, we placed the WASH results strategically just before reporting the meta-regression results, and after the regression results of the other indicators, to better contextualize the connection between these outcomes.*

**How would the limitations impact your conclusions however? I think the limitations you mentioned especially second and third are very important to consider in how your results could be affected and whether your conclusions would be changed. Consider how publication biases and not testing for interactions (e.g. poverty and population) may also impact your conclusions.**

*r/ We thank the reviewer for this insightful comment. We clarify that interactions with poverty and population were explicitly tested in our meta-regression (Table 2), serving as the primary evidence for our conclusion that research impact is possibly context-dependent. Regarding limitations such as publication bias and workforce scope, we view our findings as a conservative estimate of the globally visible scientific enterprise, which drives international policy, rather than a complete negation of local grassroots capacity. Therefore, we maintain that our conclusions regarding structural inequities in the dominant research ecosystem remain robust.*

**Generally, I recommend you avoid causative language because as you mentioned the study is observational. So in some areas where there are strong recommendations for funding allocation etc try to tone that down reminding the reader that there are caveats and other variables could be playing a role here that were not measured.**

*r/ We thank the reviewer. The modifications have been made accordingly.*

**Editorial and Data Presentation Modifications?**

**Reviewer #3**

**Line 123: Replace sanity with sanitation.**

*r/ We thank the reviewer. The change has been made.*

**Line 438: why as expected? This will be clearer if you are more explicit with your expectations in the intro.**

*r/ We thank the reviewer for this comment. We have decided to remove the phrase 'as expected' to ensure a more impartial reporting of the results.*

**Line 540: Replace larger with more populous to avoid thinking of land mass.**

*r/ We thank the reviewer. The change has been made.*

**Line 561: Change to fulfill.**

*r/ We thank the reviewer. The change has been made.*

**Please provide R package citations – the Zenodo reference lists more R packages that were used than what is shown in the manuscript.**

*r/ We thank the reviewer for this comment. We have explicitly mentioned the primary packages used for the statistical analyses (i.e., meta, metafor) in the Methods section. To maintain conciseness and avoid an excessive number of technical citations in the main text, the complete list of packages and dependencies is detailed in the fully reproducible R script available in the Zenodo repository.*

**Summary and General Comments**

**Reviewer #1**

**This is a solid, courageous, and necessary study that opens a promising line of research — assessing not only how much science is produced, but how much it truly contributes to global health. However, it represents only a first step; mixed-method approaches (quantitative + qualitative) are recommended to better understand the mechanisms of research translation and implementation.**

**I recommend few minor changes and then, in my opinion, the manuscript can be published.**

*r/ We sincerely thank the reviewer for their encouraging feedback and valuable perspective on the future of this research line. We fully agree that mixed-method approaches are the next logical step to understand translation mechanisms. We confirm that all suggested changes have been incorporated into the manuscript.*

**Reviewer #2**

**This manuscript presents an ambitious longitudinal analysis linking NTD research output to population-level health, socioeconomic, and research capacity indicators across income groups. While the study addresses an important gap in understanding research impact, several methodological and interpretive issues need attention. The paper needs recalibration to match conclusions to what the observational design can support.**

*r/ We sincerely thank the reviewer for recognizing the ambition and importance of this analysis. We confirm that the concerns regarding methodological and interpretive recalibration have been fully addressed in the revised manuscript, ensuring that all conclusions are strictly supported by the observational design.*

**1.The title, abstract, and throughout claim to measure ‘‘impact’’ and ‘‘effects’’, but the methods only establish cross-sectional correlations. The authors should consider whether this is fundamentally a study showing research-outcome correlations vary by setting (more defensible) versus research impact on outcomes (much harder to establish with this design).**

*r/ We thank the reviewer for this critical observation regarding causal language. We agree that 'impact' must be used with caution. To address this, we have modified the title to 'Estimating the Potential Impact...' to better reflect the inferential nature of the study. Furthermore, while we respectfully clarify that our design is longitudinal rather than cross-sectional, we have carefully revised the manuscript to ensure that the terminology reflects non-causal estimations of possible associations, explicitly acknowledging the inherent limitations of the observational design as detailed in the Conclusions.*

**2.The authors employ random-effects meta-analysis to synthesize regression results across income groups, which is unconventional since meta-analysis is typically designed to combine findings from independent studies rather than strata within a single dataset. While this approach is not methodologically incorrect, it requires clear justification for why meta-analysis was chosen over more standard alternatives such as mixed-effects models with income group as a random effect, stratified regression with interaction terms, or formal heterogeneity tests like the Chow test. Without this justification, readers cannot assess whether the chosen analytical framework appropriately addresses the research question or whether simpler, more transparent methods might be preferable.**

*r/ We thank the reviewer for this insightful methodological inquiry. While we agree that hierarchical mixed-effects models or stratified regressions with interaction terms are valid alternatives, we justify the use of random-effects meta-analysis because a primary objective of this study was to explicitly quantify the magnitude of between-group variability (I^2^, τ²) as a central finding, rather than merely controlling for it. This heterogeneity score provided the necessary statistical foundation for our third analytical step: meta-regression, which was specifically designed to identify the sources of this variability. By establishing the precise magnitude of heterogeneity first, we could empirically justify the investigation into moderators such as poverty and population size.*

**3.The authors correlate research publications with health indicators from the same year, which cannot demonstrate causality since research typically requires several years to translate into population health improvements. This approach only shows that countries producing more research also have better health in the same time period, but cannot determine whether research causes better health or whether healthier, wealthier countries simply produce more research. The authors must either introduce temporal lags (comparing past research with future health outcomes) or explicitly reframe their findings as correlational patterns rather than evidence of research impact.**

*r/ We thank the reviewer for this opportunity to clarify our analytical strategy. First, we respectfully distinguish our methodology from simple cross-sectional correlation analyses. Our approach utilized regression models, random-effects meta-analyses, and meta-regressions, rather than simple correlation coefficients (ranging from -1 to 1). Unlike simple correlations, these regression models allow us to estimate the potential magnitude of change (*β*) in population-level indicators associated with research output, taking into account the specific trajectories and dynamics of the last decades. This longitudinal design utilizes the entire history of available registered publications and indicator registries (up to 2024), rather than relying on a static comparison of a single year.*

*While we agree that a single paper requires time to translate into impact, the cumulative volume of research on a specific topic is expected to align with improvements in related indicators over time, under the premise that we cannot resolve health challenges that we do not study. To account for the "wealth" confounder, we explicitly stratified our models by World Bank income groups. This ensures that our results reflect associations within specific economic contexts (e.g., the relationship between research and health specifically within Low-Income Countries) rather than comparing poor countries to rich ones. Finally, we have carefully reviewed the manuscript to ensure the language reflects these "estimations of potential impact" and "data-driven associations" rather than definitive causal claims, explicitly acknowledging the observational nature of the design*

**4.Attributing research to the first author’s country is problematic because it miscounts where research actually benefits populations. For example, a US-led study on Kenyan diseases gets credited to the US, not Kenya. This systematically inflates high-income countries’ apparent contributions since they dominate first authorship even for work conducted in low-income settings. The authors’ own finding that research capacity grows in rich countries while health improvements occur in poor countries suggests this attribution method fails to track where research impact actually occurs.**

*r/ We thank the reviewer for highlighting this critical nuance regarding the attribution of research benefits. While we acknowledge that using the first author’s affiliation does not perfectly capture the geographic location of fieldwork or the direct beneficiary populations, we maintained this bibliometric standard as it serves as a robust proxy for intellectual leadership, funding origin, and agenda-setting power. By mapping the "origin" of the science to the primary affiliation, we aimed to identify which health systems are driving the global research agenda, rather than solely where the data is collected.*

*Crucially, the discrepancy the reviewer notes: that high-income countries often lead research on diseases affecting low-income settings, is not merely a methodological limitation but a central structural global gap. This "attribution gap" empirically demonstrates the lack of scientific coherence described in our Introduction, validating our conclusion that the global research ecosystem remains driven by external priorities rather than local leadership. By mapping authorship this way, we expose the structural inequity that requires the policy translation mechanisms we advocate for, particularly the strengthening of local research capacity in the countries bearing the disease burden*

**5.The Results are clearly presented statistically, but the interpretation throughout overstates what the data can show. The fundamental issue—that same-year correlations cannot demonstrate research impact—undermines the core narrative.**

*r/ We thank the reviewer for this assessment. We respectfully clarify that our analysis is not based on cross-sectional same-year correlations, but on a longitudinal regression framework spanning several years, specifically designed to capture cumulative trends rather than immediate effects. However, we fully agree that interpretation must be cautious; we have revised the manuscript to explicitly frame these findings as 'associations' and 'estimations of coherence' rather than definitive evidence of causal impact, as detailed in our revised Title and manuscript body.*

**6.The strongest findings are in WASH indicators, but many WASH improvements have occurred through infrastructure investment independent of biomedical research. The mechanism linking NTD publications to handwashing facilities remains unclear. This domain may reflect confounding by general development more than others. The authors should discuss alternative explanations for WASH improvements, clarify plausible mechanistic pathways between publications and infrastructure outcomes, and consider whether WASH indicators are appropriate measures of research impact.**

*r/ We thank the reviewer for this insightful comment. We clarify that it is not the focus of the paper to study drivers other than research impact on, for example, WASH indicators. However, the associations found do not exclude that other variables can affect the outcome; they are simply not measured in this analysis. The aim of the paper is to study estimations of potential impact between research and population-level indicators under the concept of scientific coherence: more research should ideally be translated into improvements in society. If this type of coherence does not exist, it is a relevant finding to discuss, just as if it does exist.*

**7.Did WASH improvements accelerate after NTD research increased, or did both trend upward independently? Without temporal sequencing, the authors just showing correlation.**

*r/ We thank the reviewer for this important question. We clarify that our analysis is longitudinal (spanning 2000-2024), specifically designed to track these trajectories over time rather than as a static snapshot. While we acknowledge that independent upward trends are possible, our objective was to measure scientific coherence: the extent to which research intensity aligns with improvements in related determinants. If both trend upward significantly within specific income groups, this represents the structural alignment we aimed to estimate, regardless of strict directional causality.*

**8.Current conclusion overstates what the data can support.**

*r/ We thank the reviewer for this observation. We respectfully maintain that our conclusion is conservative and strictly data-driven. We explicitly state that the hypothesis was “only partially supported” and frame the findings as potential “associations” rather than causal effects. Furthermore, the conclusion focuses heavily on the lack of consistent alignment (coherence), which is directly supported by the non-significant and heterogeneous results observed in our analysis.*

**9.The manuscript’s language is generally strong, but minor grammatical corrections (e.g., sanity to sanitation, line 132), tense consistency, and improved precision in causal wording are required for professional polish and journal compliance.**

*r/ We sincerely thank the reviewer for this careful reading. We have corrected “sanity” to “sanitation” and conducted a thorough proofreading to ensure tense consistency. Furthermore, we have refined the wording throughout the manuscript to strictly reflect non-causal associations, ensuring professional precision.*

**Reviewer #3**

**The authors should be commended for undertaking such an extensive study. I appreciate all the work that was done and the thought that went into putting this study together. I also appreciate how they provided the data and the R code. I do have some significant concerns however that may be able to be addressed through providing the reader with more justification in the methods and intro although some additional data analyses and visualizations will be necessary.**

*r/ We sincerely thank the reviewer for their encouraging comments. We confirm that all proposed modifications regarding methodological justifications, additional analyses, and visualizations have been fully addressed in the revised manuscript.*

**The path from pubs (research output) to policy/regulations that impact population health outcomes is more complicated than what is implied here and there are likely significant time lags from research to population health outcomes that have not been considered. The assumption that research predominantly drives health gains and that countries with more NTD pubs will have better health metrics needs to be fleshed out more to be convincing.**

*r/ We thank the reviewer for this insightful comment. We fully acknowledge the complexity and time lags inherent in translating research into policy; this is precisely why we employed a longitudinal design to capture cumulative associations over decades rather than immediate effects.*

*We clarify that while other unmeasured drivers undoubtedly exist, our aim is to test the specific hypothesis of scientific coherence: that increased research volume should ideally translate into improvements in society. If this type of coherence does not exist, or is obscured by the complexities the reviewer mentions, it is a relevant structural finding to discuss, just as if it does exist.*

**Although I appreciate the complicated statistical methods, I am not convinced of the conceptual approach here. The authors state that HIC countries already produce most of the NTD research but what about the ones doing so in collaboration with lower income groups to address their problems? The authors assign country origin based on where the first author of the paper is from but I’m not sure this accounts for co-authors that could be from lower income countries (and why not senior author for example). I will need stronger justification in methods and intro.**

*r/ We thank the reviewer for this important conceptual challenge. We justify using the first author as the primary proxy because this position typically reflects the institution driving the immediate research agenda. While we recognize the value of co-authorship, the fact that High-Income Countries often dominate this primary position, even within collaborations involving Low-Income Countries, is precisely one of the structural inequities we discussed. We have strengthened this justification in the Methods section.*

**Furthermore, I’m not convinced that aggregating all NTDs together (from parasitic to viral diseases) and looking at weighted pub numbers as a response or predictor variable indicative of research output is a suitable approach. Some NTDs receive more attention than others, and some are more prevalent in one income group than the other so I am concerned as to whether a lot of important signals are being muddled because NTDs are aggregated. For example what if there are clear signals for malaria but not for snake bites and by aggregating everything together you are mixing signals? I think for me to be convinced of this approach I will need stronger justification in the methods and intro as to why this approach is suitable.**

*r/ We thank the reviewer for this profound epistemological challenge. We defend our aggregation strategy because our objective is to test the systemic hypothesis of scientific coherence: the postulate that the institution of science, as a cumulative force, drives development, rather than to evaluate disease-specific epidemiology. From a meta-research perspective, NTDs function not merely as a biological list, but as a unified policy and funding construct defined by the WHO Roadmap. Therefore, analyzing them as an aggregate is necessary to test whether this global policy framework serves as a reliable mechanism for health improvement.*

*If we were to disaggregate, we would merely observe well-known biological differences (e.g., Malaria receives more funding than snakebite). By aggregating, we filter out this biological variance to isolate the structural signal of scientific capacity and institutional attention. Consequently, if strong signals for some diseases are diluted by weak signals for others, this does not muddle our conclusion; rather, it empirically falsifies the generalizability of the science-equals-health narrative. It proves that the current research ecosystem does not function as a coherent system, but as a fragmented one, a structural finding that can only be observed when the system is analyzed as a whole.*

**I'm also unclear as to why the conclusions may not be a mere reflection of multiple factors beyond pubs where over time the global society has improved public health measures. For example, factors other than number of pubs (e.g. word of mouth etc) may have improved sanitation and hygiene over time and positive associations indicate that in more recent years we have more pubs but we also have more community awareness (independent of pubs) that happens to correlate with more hygiene and sanitation. If this was addressed in the paper I was not able to catch it and thus may need more explicit explanation. Because the authors state that NTD research has expanded over the last two decades, could they look how the population health metrics have changed before and after this large expansion in literature? Unless the expansion is less drastic than that. Because otherwise, why go back to the literature from the early 1900s?**

*r/ We thank the reviewer for this insightful critique regarding secular trends and the timeframe of our analysis. First, we respectfully clarify a distinction in our methodology: while we retrieved bibliometric data back to 1904 to accurately calculate the cumulative stock of historical knowledge, our statistical regression analysis and meta-regressions were strictly limited to the 2000–2024 period, allowing us to test the associations during the period of maximum growth the reviewer alludes to.*

*Regarding the concern that improvements may simply reflect general societal progress (e.g., community awareness) rather than research impact, we argue that it is not the focus of the paper to study drivers other than research impact on population-level indicators. However, the associations found do not exclude that other variables can affect the outcome; they are simply not measured in the primary analysis. The aim of the paper is to study estimations of potential impact between research and population-level indicators under the concept of scientific coherence: more research should ideally be translated into improvements in society. If this type of coherence does not exist, it is a relevant finding to discuss, just as if it does exist. Furthermore, the influence of other structural variables on the base model was explicitly assessed in the meta-regression analysis.*
